# Dr. William A. Oliver

**Published:** 1915-06

**Authors:** 


**Dr. William A. Oliver,** Buffalo 1884, died at the Canandaigua Hospital, April 6, aged 58. He was a practitioner of Penn Yan.

